# The role of p53 in the DNA damage-related ubiquitylation of S2P RNAPII

**DOI:** 10.1371/journal.pone.0267615

**Published:** 2022-05-05

**Authors:** Barbara N. Borsos, Vasiliki Pantazi, Zoltán G. Páhi, Hajnalka Majoros, Zsuzsanna Ujfaludi, Ivett Berzsenyi, Tibor Pankotai

**Affiliations:** Institute of Pathology, Albert Szent-Györgyi Medical School, University of Szeged, Szeged, Hungary; Augusta University, UNITED STATES

## Abstract

DNA double-strand breaks are one of the most deleterious lesions for the cells, therefore understanding the macromolecular interactions of the DNA repair-related mechanisms is essential. DNA damage triggers transcription silencing at the damage site, leading to the removal of the elongating RNA polymerase II (S2P RNAPII) from this locus, which provides accessibility for the repair factors to the lesion. We previously demonstrated that following transcription block, p53 plays a pivotal role in transcription elongation by interacting with S2P RNAPII. In the current study, we reveal that p53 is involved in the fine-tune regulation of S2P RNAPII ubiquitylation. Furthermore, we emphasize the potential role of p53 in delaying the premature ubiquitylation and the subsequent chromatin removal of S2P RNAPII as a response to transcription block.

## Introduction

DNA double-strand breaks (DSBs) are the most deleterious lesions, thus the fine-tuning of the related repair processes is indispensable to preventing genome instability. Ataxia-telangiectasia mutated (ATM) kinase and DNA-dependent protein kinase (DNA-PK) are principal regulators in the precise coordination of the two main subpathways of DSB repair, homologous recombination (HR) and non-homologous end joining (NHEJ), respectively [1–4]. Although ATM and DNA-PK are responsible for the activation of different repair pathways, they have common target proteins, such as H2A.X and p53 [5–9]. Following DNA damage, ATM and DNA-PK phosphorylate p53 at Ser15, resulting in its activation and nuclear accumulation [6–9].

p53 is a well-known tumor suppressor whose function during transcription was thought to be restricted to the initiation phase. However, a novel role of p53 in transcription elongation has been recently revealed in yeast and human models [10–14]. We demonstrated for the first time in human cells that p53 binds to non-sequence specific gene regions, which is further enhanced following Actinomycin D (ActD)-induced transcription block. Nonetheless, a significant decrease in RNA polymerase II (RNAPII) occupancy was detected upon ActD treatment, and interaction was established between p53 and the elongating RNAPII (S2P RNAPII), suggesting a possible role of p53 in the DNA damage-related dislodgement of RNAPII. The remission in the S2P RNAPII level was proved to be the consequence of its proteasomal-mediated degradation. Moreover, p53 was found to co-localize with γH2A.X at the damage foci, which suggested a potential role of p53 in transcription silencing to allow the recruitment of DNA repair factors to the damage site [13].

Depending on the severity of DNA damage, the fate of the stalled RNAPII can vary [15–17]. Cockayne syndrome group B (CSB) and Transcription factor II H (TFIIH) participate in the forward (at minor lesions) and reverse (at bulky lesions) translocation of RNAPII, respectively [18, 19]. However, upon severe DNA damage, the permanently stalled S2P RNAPII is marked for polyubiquitylation-mediated proteasomal degradation to promote the efficient repair mechanism [20, 21]. Following DNA damage, RNAPII remains phosphorylated at Ser2 of the C-terminal domain to prevent the initiation of a new transcription cycle and to allow its ubiquitylation-related removal [22]. Several E3 ligase complexes are involved in the DNA damage-related ubiquitylation of S2P RNAPII, such as Neural precursor cell expressed developmentally down-regulated protein 4 (NEDD4), Breast cancer 1 (BRCA1)–BRCA1-associated RING domain protein 1 (BARD1), and ElonginA/B/C–Cullin-5–RING-box protein 2 (EloA/B/C–CUL5–RBX2) [23–26]. WW domain-containing protein 2 (WWP2) has been identified hitherto as an interaction partner of RNAPII, and as a key E3 ligase in the DSB-related polyubiquitylation of RNAPII [27, 28]. Moreover, DNA-PK has been shown to have an essential role in transcription silencing by facilitating the WWP2-dependent ubiquitylation of S2P RNAPII and the recruitment of the 26S proteasome to the break site [17, 27, 29].

Here, we shed light on the potential involvement of p53 in the polyubiquitylation of S2P RNAPII following ActD-induced transcription block. Furthermore, we found that p53 negatively affects the precocious removal of S2P RNAPII from actively transcribed gene regions to ensure time for the proper repair process, suggesting an auxiliary role of p53 in transcription elongation.

## Materials and methods

### Cell cultures

HCT116 p53+/+ and p53-/- (kindly provided by Prof. Bert Vogelstein, John Hopkins University, Baltimore, MD) isogenic colorectal carcinoma cell lines were used for the experiments [30, 31]. HCT116 cells were grown in high glucose DMEM (Dulbecco’s Modified Eagle Media; Lonza) supplemented with 8 mM glutamine (Sigma-Aldrich), 1x antibiotic–antimycotic solution (Sigma-Aldrich), and 10% fetal bovine serum (FBS; Lonza). U2OS cells were maintained in low glucose DMEM (Dulbecco’s Modified Eagle Media; Lonza) supplemented with 4 mM glutamine (Sigma-Aldrich), 1x antibiotic–antimycotic solution (Sigma-Aldrich), and 10% fetal bovine serum (FBS; Lonza). Cells were maintained at 37°C in humidified atmosphere with 5% CO_2_. Our cell culture-related study had been approved to be performed in the University of Szeged according to the TMF/43-18/2015 decision before the study began.

### Actinomycin D treatment

200 nM (U2OS cells) or 400 nM (HCT116 cells) Actinomycin D (ActD) (Sigma-Aldrich) was used to arrest transcription elongation at different time-points.

### Chromatin immunoprecipitation (ChIP)

Cells were fixed with 1% formaldehyde (Sigma-Aldrich) for 10 min, then fixation was halted with 125 mM glycine (Sigma-Aldrich). Cells were centrifuged at 2,000 rpm for 5 min at 4°C, then were resuspended in cell lysis buffer (5 mM PIPES pH 8.0, 85 mM KCl, 0.5% NP-40; Sigma-Aldrich) complemented with 1xPIC (Protease Inhibitor Cocktail; Roche), and incubated on ice for 10 min. Pellets were depleted with 5 min centrifugation at 2,000 rpm, 4°C, resuspended in nuclear lysis buffer (50 mM Tris-HCl pH 8.0, 10 mM EDTA pH 8.0, 0.8% SDS; Sigma-Aldrich) supplemented with 1xPIC (Roche), and incubated on ice for 1 h. Chromatins were sheared 4x 20 sec ON/ 1 min OFF with Bioruptor Pico sonicator (Diagenode), then diluted four times with dilution buffer (10 mM Tris-HCl pH 8.0, 0.5 mM EGTA pH 8.0, 1% Triton X-100, 140 mM NaCl; Sigma-Aldrich) complemented with 1xPIC (Roche). 30 μg chromatin samples were pre-cleared with 4 μl Sheep anti-Rabbit IgG Dynabeads (Novex) for 2 h rotation at 4°C. Pre-cleared chromatin samples were incubated with 2 μg anti-S2P RNAPII (Abcam, ab5095) antibody overnight rotating at 4°C. Chromatin–antibody complexes were captured overnight rotating with 40 μl Sheep anti-Rabbit IgG Dynabeads (Novex). Subsequently to several washing steps, chromatin–antibody complexes were eluted and precipitated. Pellets were resuspended in TE buffer (10 mM Tris-HCl pH 8.0, 1 mM EDTA pH 8.0; Sigma-Aldrich) and reverse-crosslinked. The desired DNA fragments were purified with phenol–chloroform extraction then precipitated with absolute ethanol. Pellets were dissolved in TE buffer [32]. Occupancy of S2P RNAPII was monitored with qPCR (Thermo PikoReal 96 Real-Time PCR system; Thermo Fisher Scientific). Sequences of primers used for qPCR are listed in S1 Table.

qPCR quantification was performed by using a TIC (total input control) standard curve. The precipitated amount of DNA in each sample was normalized to the amount of DNA in the NAC (no antibody control).

### Western blot

HCT116 p53+/+, HCT116 p53-/-, and U2OS cells were harvested in lysis buffer (150 mM NaCl, 1% Triton X-100, 50 mM Tris-HCl pH 8.0; Sigma-Aldrich) supplemented with 1xPIC (Roche), 20 μM PR-619 DUBi (Deubiquitylase Inhibitor; Calbiochem), and 1x PhosSTOP (Roche) on ice for 10 min, then sonicated 10x 30 sec ON/ 30 sec OFF in Bioruptor Pico sonicator (Diagenode). Protein concentration was measured with Pierce^TM^ BCA Protein Assay Kit (Thermo Fisher Scientific), then 30 μg protein lysates were mixed with NuPAGE^TM^ LDS Sample Buffer (4x) (Thermo Fisher Scientific) and boiled for 10 min. Proteins were separated in pre-casted Bolt^TM^ 4–12% Bis-Tris Plus gradient gels (Thermo Fisher Scientific). 1x NuPAGE^TM^ MOPS SDS Running Buffer (Thermo Fisher Scientific) and 1x NuPAGE^TM^ Transfer Buffer (Thermo Fisher Scientific) were used for SDS-PAGE and transfer, respectively. Proteins were transferred onto Amersham Hybond ECL-nitrocellulose membrane (GE Healthcare). Unspecific binding sites of the membranes were blocked with 5% non-fat dry milk–TBST (Tris-Buffered Saline complemented with Tween 20), then the membranes were incubated with primary [S2P RNAPII ab5095 (Abcam) 1:4000, p53 MA5-12557 (Thermo Fisher Scientific) 1:1000, GAPDH MAB374 (Merck–Millipore) 1:1000], and horseradish peroxidase (HRP)-conjugated secondary antibodies [GAR-HRP IgG P0448 (Dako), RAM-HRP IgG P0260 (Dako)]. Chemiluminescent detection was conducted using Immobilon Western Chemiluminescent HRP substrate (Merck–Millipore) and G:BOX Chemi XRQ (Syngene) system.

### Tandem ubiquitin-binding entities (TUBEs) assay

TUBE2 (UM402; LifeSensors) agarose beads were used to capture polyubiquitin moieties from HCT116 p53+/+, HCT116 p53-/-, and U2OS cell lysates. Cells were harvested in TENT buffer (50 mM Tris-HCl pH 8.0, 2 mM EDTA pH 8.0, 150 mM NaCl, 1% Triton X-100; Sigma-Aldrich) complemented with 1xPIC (Roche), 20 μM PR-619 DUBi (Calbiochem), and 1x PhosSTOP (Roche), incubated on ice for 10 min, then sonicated 12x 30 sec ON/ 30 sec OFF in Bioruptor Pico sonicator (Diagenode). Afterwards, samples were centrifuged at 14,000 g for 10 min at 4°C. Protein concentration of the supernatants was measured with Pierce^TM^ BCA Protein Assay Kit (Thermo Fisher Scientific). 20 μl TUBEs beads/ IP were washed twice with TBST (20 mM Tris-HCl pH 8.0, 150 mM NaCl, 0.1% Tween 20; Sigma-Aldrich) complemented with 1xPIC (Roche), 20 μM PR-619 DUBi (Calbiochem), and 1x PhosSTOP (Roche). Between each washing step, beads were centrifuged at 1,000 g for 1 min at 4°C. 1.5 mg protein was added to 20 μl TUBEs beads and exceeded with TENT + inhibitors up to 500 μl final volume, then samples were rotated for 2 h at 4°C. Beads–polyubiquitin complexes were washed three times with TBST + inhibitors, then eluted in 40 μl final volume of the mixture of TENT + inhibitors and NuPAGE^TM^ LDS Sample Buffer (4x) (Thermo Fisher Scientific) at 100°C for 10 min.

### siRNA silencing

Human p53 siRNA pool (L-003329-00-0005) was used for silencing p53 in U2OS cells. As a negative control, we used non-targeting siRNA pool (D-001810-10-05). siRNA pools were ordered from Dharmacon (Thermo Fisher Scientific) and transfected to the cells using INTERFERin (Polyplus) transfection reagent and antibiotic-free DMEM medium following the manufacturer’s recommendations.

### Statistics

Statistical analyses were performed to show significant differences between ChIP datasets with one-way ANOVA after checking the normal distribution of the data in IBM SPSS 28.0. Western blots were quantified by using Fiji Image J.

## Results

### p53 delays the removal of S2P RNAPII from actively transcribed gene regions as a response to transcription block

**Fig 1:**
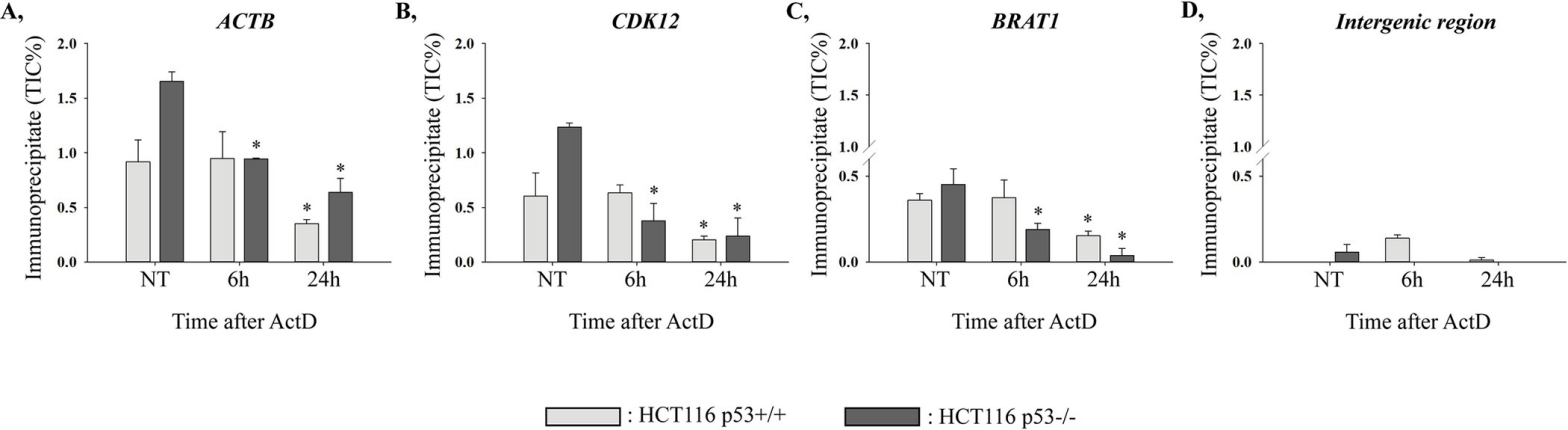
p53 affects the profile changes of elongating RNA polymerase II (S2P RNAPII) at transcriptionally active gene regions following Actinomycin D (ActD)-induced transcription elongation block. (**A–C**) S2P RNAPII occupancy was monitored with ChIP–qPCR at *ACTB*, *CDK12*, and *BRAT1* gene bodies in the presence (light grey columns; HCT116 p53+/+ cell line) and in the absence (dark grey columns; HCT116 p53-/- cell line) of p53. The profile changes were tracked under physiological conditions (NT) as well as following 6 h and 24 h ActD treatments. (**D**) Primers designed to an intergenic region were used as the negative control of the ChIP. The figure shows the representative result of one out of two independent experimental replicates. qPCR reactions were performed in duplicates. Asterisks represent statistical significance (*P ≤ 0.05) between the mean values. Mean values of ActD-treated samples were compared to the mean value of the corresponding non-treated sample by one-way ANOVA in case of each cell line.

To study whether p53 plays a role in the chromatin dislodgement of the arrested elongating RNA polymerase II (S2P RNAPII) following DNA double-strand break (DSB) induction, we monitored the profile changes of the S2P RNAPII by chromatin immunoprecipitation (ChIP) in HCT116 p53+/+ and p53-/- colorectal cancer cell lines. For this, we treated both cell lines with the transcription elongation blocking agent ActD for 6 h and 24 h. The occupancy of S2P RNAPII was tracked at a certain gene body region of three actively transcribed genes, *ACTB*, *CDK12*, and *BRAT1* (Fig 1A–1C, respectively). Based on our previously published results, *ACTB*, *CDK12*, and *BRAT1* can be classified into distinct clusters according to their transcription profile and S2P RNAPII occupancy detected on these genes [13]. As a negative control, we used primers for an intergenic region, where active transcription does not take place (Fig 1D).

Under physiological conditions, the level of S2P RNAPII at the gene body of *ACTB*, *CDK12*, and *BRAT1* is higher in HCT116 p53-/- cells compared to p53+/+, which supports the restraining role of p53 in the normal transcription elongation process (Fig 1A–1C). In HCT116 p53+/+ cells, the occupancy of S2P RNAPII is not altered at any of the examined genes following 6 h ActD treatment, while a dramatic decrease is observed at 24 h ActD on these transcribed regions. On the contrary, in HCT116 p53-/- cells, a significant attenuation is detected on all three desired genes already upon 6 h ActD which is further reduced following 24 h ActD treatment (Fig 1A–1C). These data are supported by two independent ChIP experiments performed in HCT116 p53+/+ and p53-/- cells under the same conditions (Fig 1 and S1 Fig).

Conclusively, in HCT116 p53-/- cells, the S2P RNAPII occupancy is significantly reduced already at 6 h, while in HCT116 p53+/+ cells, this attenuation is observed only after 24 h ActD treatment. In HCT116 p53+/+ cells, the binding of S2P RNAPII still remains relatively high at 6 h, which denotes that in the presence of p53, the stalled S2P RNAPII cannot be removed until this time-point (Fig 1A–1C). These results reveal that in colorectal carcinoma cells, p53 delays the removal of S2P RNAPII from actively transcribed regions as a response to transcription elongation block-induced DNA damage.

### p53 is involved in the regulation of S2P RNAPII ubiquitylation following ActD-induced transcription block

**Fig 2:**
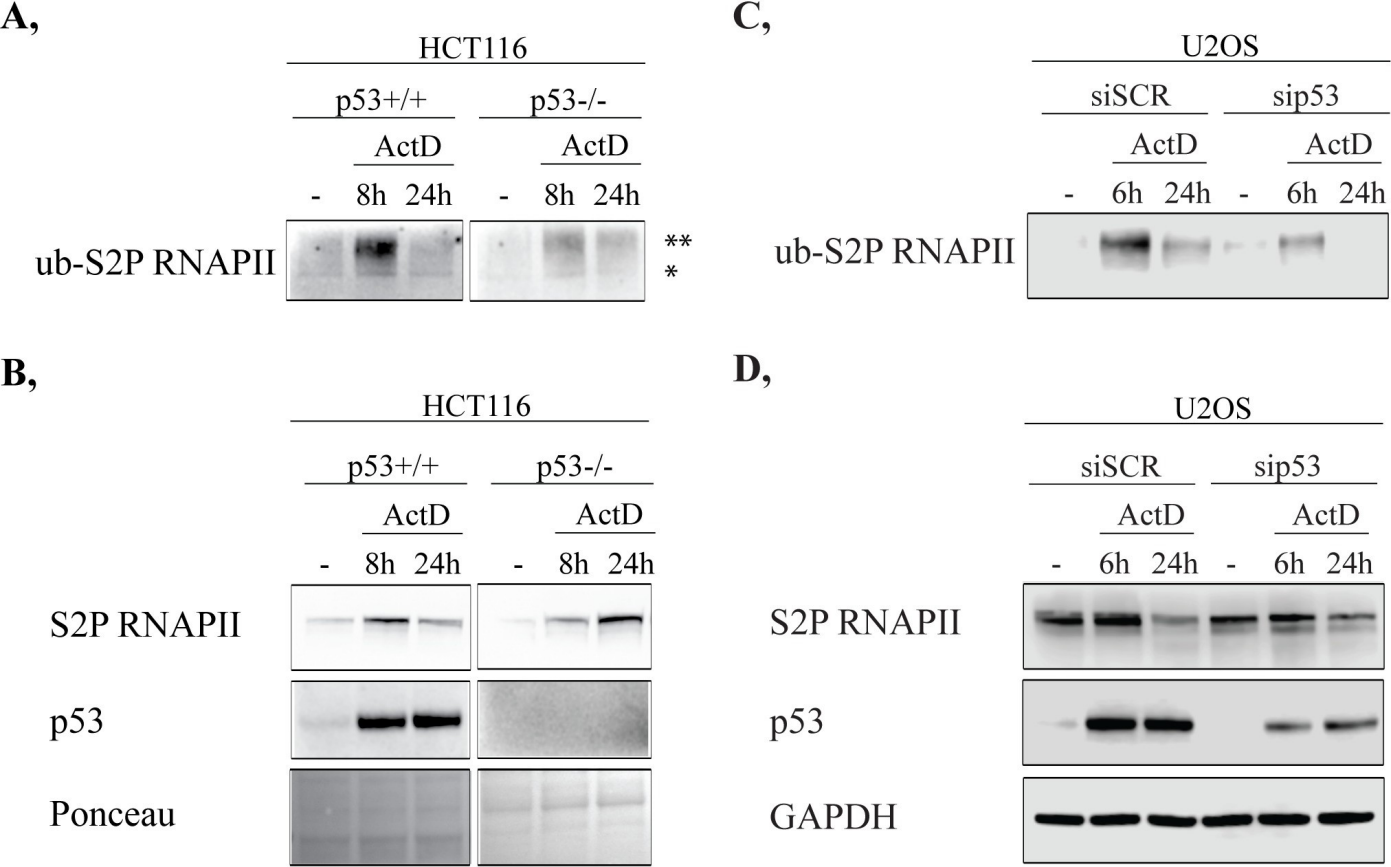
p53 involvement in the ubiquitylation of RNAPII following ActD treatment is a ubiquitous process. (**A**) Tandem ubiquitin-binding entities (TUBEs) pull-down followed by Western blot detection was performed in HCT116 p53+/+ (left panel) and p53-/- (right panel) cells. TUBEs experiment was accomplished under physiological conditions as well as 8 h and 24 h following ActD treatments. From the pulled-down ubiquitylated protein pool, polyubiquitylated S2P RNAPII (ub-S2P RNAPII) was detected with anti-S2P RNAPII antibody. *: monoubiquitylated S2P RNAPII, **: polyubiquitylated S2P RNAPII (**B**) Western blot experiment on whole cell lysates of HCT116 p53+/+ (left panel) and p53-/- (right panel), which were used for TUBEs pull-down assay. Total protein level changes of S2P RNAPII and p53 following 8 h and 24 h ActD treatments were immunodetected using specific antibodies. Ponceau staining was applied to detect the equal loading of the input samples. (**C**) Tandem ubiquitin-binding entities (TUBEs) pull-down followed by Western blot detection was performed in non-targeting and p53 silencing siRNA-transfected (referred to as siSCR and sip53, respectively) U2OS cells. In U2OS cells, TUBEs assay was performed under physiological conditions, 6 h and 24 h following ActD treatments. From the pulled-down ubiquitylated protein pool, polyubiquitylated S2P RNAPII (ub-S2P RNAPII) was detected with anti-S2P RNAPII antibody. (**D**) Western blot experiment on whole cell lysates of U2OS cells which were used for TUBEs pull-down assay. Total protein level changes of S2P RNAPII and p53 as a response to 6 h and 24 h ActD treatments were immunodetected using specific antibodies. GAPDH detection was applied to detect the equal loading of the input samples.

Following severe DNA damage, the elongating form of RNAPII is assigned to ubiquitylation-mediated proteasomal degradation to allow access for the recruitment of repair factors [17, 20]. Using HCT116 p53+/+ and p53-/- cell lysates, we pulled-down the ubiquitylated protein pool by tandem ubiquitin-binding entities (TUBEs). Subsequently, with immunoblot, we examined the ubiquitylated S2P RNAPII (ub-S2P RNAPII) pool in basal conditions as well as following 8 h and 24 h ActD treatments. Ub-S2P RNAPII is detected mainly at 8 h ActD treatment, while at 24 h the majority of the ub-S2P RNAPII pool has been presumably degraded (Fig 2A and S2 Fig).

In HCT116 p53+/+ cells, the total protein level of S2P RNAPII is increased at 8 h ActD, then it returns to basal level after 24 h (Fig 2B and S2 Fig). On the contrary, in HCT116 p53-/- cells the S2P RNAPII still remains accumulated after 24 h ActD treatment (Fig 2B and S2 Fig).

Additionally, we investigated the changes in the protein level of p53 upon ActD (Fig 2B and S2 Fig). We detected an increase in the protein level of the total p53 pool following transcription elongation block (Fig 2B and S2 Fig).

To verify whether the p53-mediated ubiquitylation is specific for HCT116 cell line or it is a general phenomenon, we performed TUBEs assay on U2OS cells (Fig 2C and S3 Fig). We depleted the p53 with siRNA transfection and as a control, we used non-targeting siRNA (referred to as sip53 and siSCR, respectively). Subsequently, 6 h and 24 h of ActD treatments were applied and the ubiquitylated protein pool was captured by TUBEs pull-down, then changes in the level of ub-S2P RNAPII were detected by Western blot (Fig 2C and S3 Fig). In siSCR-transfected U2OS cells, following 6 h ActD treatment, a relatively high amount of ub-S2P RNAPII is observed, which is decreased 24 h after ActD treatment (Fig 2C and S3 Fig). Similar kinetics, but less ub-S2P RNAPII is detected in sip53-transfected samples (Fig 2C and S3 Fig). Furthermore, following 24 h ActD treatment, the total protein level of S2P RNAPII remained accumulated in sip53-transfected cells, while in mock siRNA-transfected cells it was diminished by that time point (Fig 2D and S3 Fig). The efficiency of p53 silencing was verified by Western blot using p53-specific antibody (Fig 2D and S3 Fig). These results support our previous TUBEs assay performed on HCT116 p53+/+ and p53-/- cells, advocating a ubiquitous role of p53 in the ubiquitylation of S2P RNAPII as a response to transcription block.

These data suggest a pivotal role of p53 in the ubiquitylation of S2P RNAPII following transcription elongation arrest. In the absence of p53, the chromatin removal of S2P RNAPII is observed already at 6 h ActD (seen in Fig 1A and 1B), while at 6 h (in U2OS cells) or at 8 h (in HCT116 cells) much less polyubiquitylated S2P RNAPII is detected (Fig 2A and S2 Fig; Fig 2C and S3 Fig), which suggests its premature ubiquitylation at earlier time-points. In conclusion, following transcription elongation block, p53 presumably fine-tune regulates the polyubiquitylation and the subsequent proteolysis of S2P RNAPII.

### p53 hinders the premature ubiquitylation of S2P RNAPII following transcription elongation arrest

**Fig 3:**
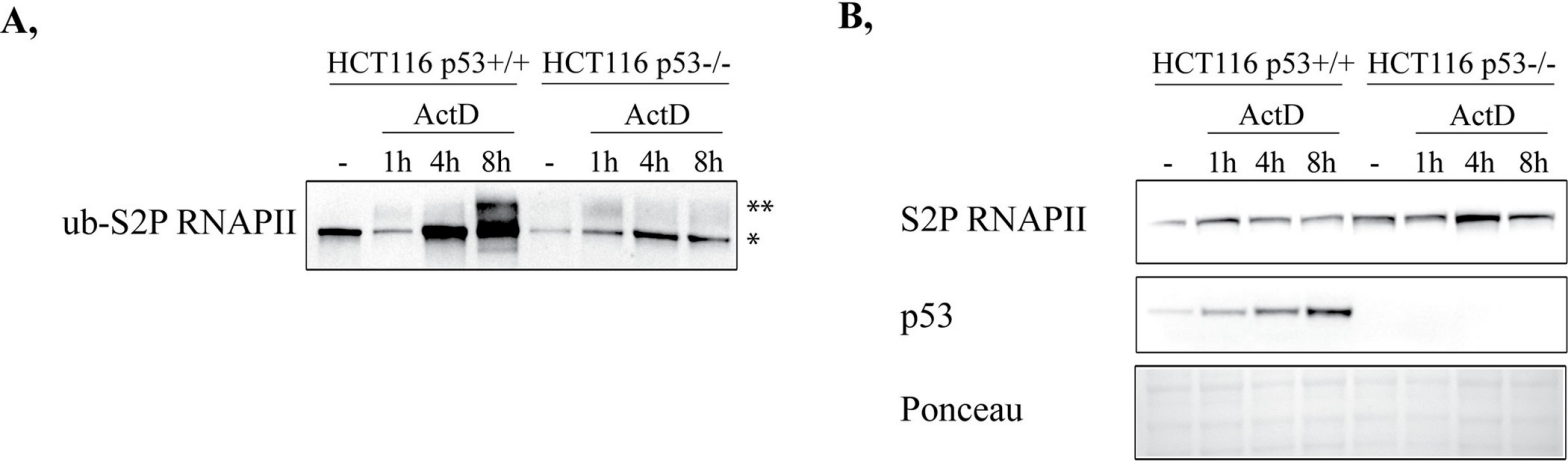
p53 is essential for preventing the premature ubiquitylation of RNAPII following ActD treatment. (**A**) Tandem ubiquitin-binding entities (TUBEs) pull-down, followed by Western blot detection was performed in HCT116 p53+/+ and p53-/- cells. TUBEs experiment was accomplished under basal conditions as well as 1 h, 4 h, and 8 h following ActD. From the pulled-down ubiquitylated protein pool, polyubiquitylated S2P RNAPII (ub-S2P RNAPII) was detected with anti-S2P RNAPII antibody. *: monoubiquitylated S2P RNAPII, **: polyubiquitylated S2P RNAPII. (**B**) Western blot experiment on whole cell lysates of HCT116 p53+/+ and p53-/-, which were used for TUBEs pull-down assay. Total protein level changes of S2P RNAPII and p53 following 1 h, 4 h, and 8 h ActD treatments in HCT116 cells were immunodetected using specific antibodies. Ponceau staining was applied to detect the equal loading of the input samples.

To reveal whether p53 indeed modulates the premature ubiquitylation of S2P RNAPII, we also included earlier time-points (1 h and 4 h) following ActD treatment at which we investigated the amount of ub-S2P RNAPII. For this, we performed TUBEs assay in 1 h, 4 h, and 8 h ActD-treated as well as in non-treated HCT116 p53+/+ and p53-/- cell lysates, and by a subsequent immunoblot detection, we compared the level of polyubiquitylated S2P RNAPII among these samples (Fig 3A and S4 Fig). In HCT116 p53+/+ cells, we detected elevated level of polyubiquitylated S2P RNAPII following 8 h ActD treatment, while in p53-/- cells much less polyubiquitylated S2P RNAPII is observed, supporting our previous results represented in Fig 2A and 2C (Fig 3A and S4 Fig). Intriguingly, at 1 h and 4 h post-ActD treatments, only a limited amount of polyubiquitylated S2P RNAPII can be seen in the presence of p53 (Fig 3A and S4 Fig). On the contrary, in the absence of p53, as a response to 1 h ActD treatment, a relatively high amount of polyubiquitylated S2P RNAPII is detected, which supports our hypothesis that p53 plays a potentially negative role in the premature ubiquitylation of S2P RNAPII (Fig 3A and S4 Fig). Therefore, we believe that p53 postpones the turnover of S2P RNAPII from the damaged chromatin to ensure time for the proper DNA repair. Monoubiquitylated S2P RNAPII (lower lane) is detected in each sample, including the basal conditions, as it is a crucial step even for the normal transcription cycle, which does not necessarily result in proteasomal degradation. Furthermore, monoubiquitylation is indispensable for the further polyubiquitylation of S2P RNAPII, thus its level is relatively high under stress conditions represented in Fig 3A.

Additionally, we monitored the alterations in the protein level of S2P RNAPII and p53 in the input samples of the TUBEs pull-down (Fig 3B and S4 Fig). In HCT116 p53+/+ cells, S2P RNAPII protein level was increased following 1 h, 4 h, and 8 h ActD treatments (Fig 3B and S4 Fig). In HCT116 p53-/- cells, a significant accrual is detected at 4 h (Fig 3B and S4 Fig). As the polyubiquitylated S2P RNAPII has been already removed by that time-point, it cannot be the ub-S2P RNAPII pool, but it rather refers to an increased rate of *de novo* transcription during the repair process. In HCT116 p53+/+ cells, the level of p53 was getting increased in a time-dependent manner following ActD treatment (Fig 3B and S4 Fig).

Accordingly, in colorectal carcinoma cells, we shed light on a hindering role of p53 in the premature ubiquitylation of S2P RNAPII following transcription elongation block, therefore protecting it from preliminary degradation to facilitate the proper DNA repair process.

## Discussion

Here, we shed light on a yet to be characterized, emerging role of p53 in the ubiquitylation of S2P RNAPII upon transcription arrest. Following transcription block, p53 delays the chromatin removal of the stalled elongating RNA polymerase II (S2P RNAPII) by stimulating its polyubiquitylation. It is an essential step for slowing down the process of subsequent proteolysis, thereby presumably ensuring the proper DNA repair.

According to the type of DNA damage and the subsequent fate of RNAPII, various ubiquitylation steps may take place [20]. We previously demonstrated in U2OS cells, that the reduction in S2P RNAPII level following ActD treatment is the consequence of its proteasomal degradation, which requires its pre-ubiquitylation [13]. However, the exact proteins [including E3 ligases, deubiquitylases (DUBs) and scaffold proteins] involved in the resolution of transcription elongation block have yet to be identified in human. E3 ligases involved in the ubiquitylation of RNAPII act synergistically to ensure the most beneficial outcome, which facilitates the recruitment of repair factors to the damage site [2]. Polyubiquitylation is a complex process, in which the linkage type determines the destiny of RNAPII. Neural precursor cell expressed developmentally down-regulated protein 4 (NEDD4) was shown to be one of the key E3 ligases responsible for the monoubiquitylation of RNAPII, and also for its subsequent K63-linked polyubiquitin chain extension [33]. Afterwards, this chain can be trimmed by Ubiquitin carboxyl-terminal hydrolase 2 (Ubp2) (shown in yeast), and eventually processed to K48-linked polyubiquitylation catalyzed by ElonginA/B/C–Cullin*-*5–RING-box protein 2 (EloA/B/C–CUL5–RBX2) and Von Hippel-Lindau/ElonginB/C–Cullin-2–RING-box protein 1 (VHL/EloB/C–CUL2–RBX1) complexes [20, 33, 34]. K48-linked chains can also be cropped by Ubiquitin carboxyl-terminal hydrolase 3 (Ubp3) (shown in yeast), hence rescuing RNAPII from subsequent degradation [35]. WWP2 E3 ligase was previously identified as an interaction partner of RNAPII, and shown to be involved in the K48-linked polyubiquitylation of RNAPII as a response to DNA damage [27, 28]. DNA-PK was shown to be necessary for WWP2 and the proteasome recruitment to the damage sites [27]. However, it still remains elusive how DNA-PK can trigger WWP2 and the 26S proteasome to the damage site. A possible explanation of this phenomenon is that a third, still unidentified protein may be involved in this process, which is presumably phosphorylated by DNA-PK.

In this study, we demonstrate that the limited amount of ub-S2P RNAPII detected in the absence of p53 following 8 h ActD treatment is not the consequence of failure in S2P RNAPII ubiquitylation, but rather a negative regulatory role of p53 in the premature ubiquitylation-related chromatin removal of S2P RNAPII. These data are supported by our chromatin immunoprecipitation (ChIP) results, in which we established that following ActD treatment p53 presence gives rise to a shift in the dwell time of the arrested S2P RNAPII at transcriptionally active gene regions, which might contribute to a more precise DNA repair process. The result of a faster removal of the stalled S2P RNAPII in the absence of p53 could lead to a precocious resolution of transcription block, which might not provide enough time for the precise DNA repair process.

In conclusion, we emphasize the potential involvement of p53 in the ubiquitylation of S2P RNAPII following transcription elongation arrest.

## Supporting information

S1 Data (XLSX)

S1 Table Primers used for ChIP–qPCR. (TIF)

S1 Fig Repetition of ChIP experiment represented in Fig 1. (TIF)

S2 Fig Relative density of Western blots represented in Fig 2. (TIF)

S3 Fig Relative density of Western blots represented in Fig 2. (TIF)

S4 Fig Relative density of Western blots represented in Fig 3. (TIF)

S5 Fig (PDF)
