# Supplementary figures and images for: The role of p53 in the DNA damage-related ubiquitylation of S2P RNAPII
Source: PLoS One. 2022 May 5;17(5):e0267615. doi: 10.1371/journal.pone.0267615 (PMC9070946; doi:10.1371/journal.pone.0267615)

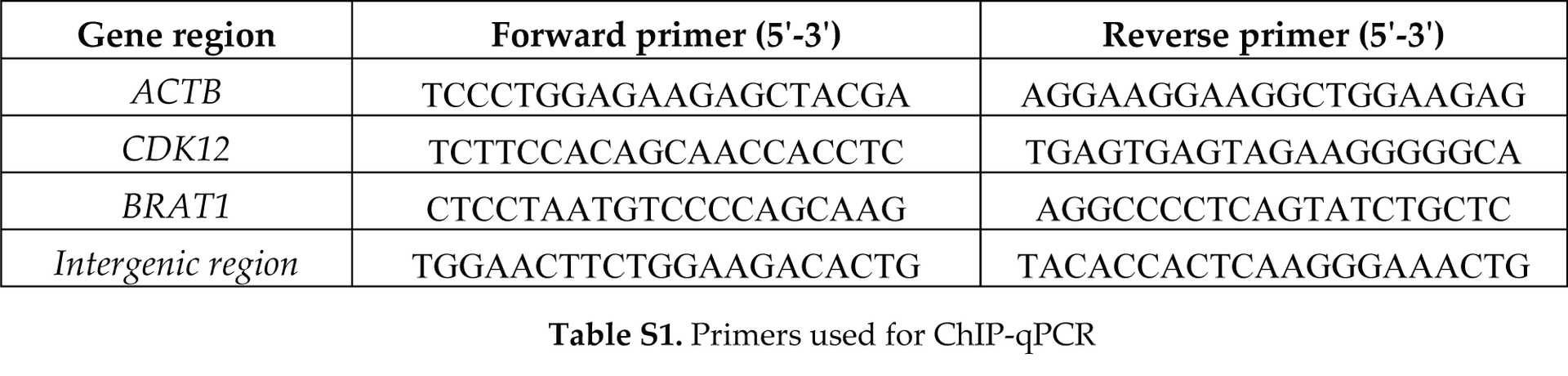

Supplement: S1 Table — (TIF) [file pone.0267615.s002.tif]

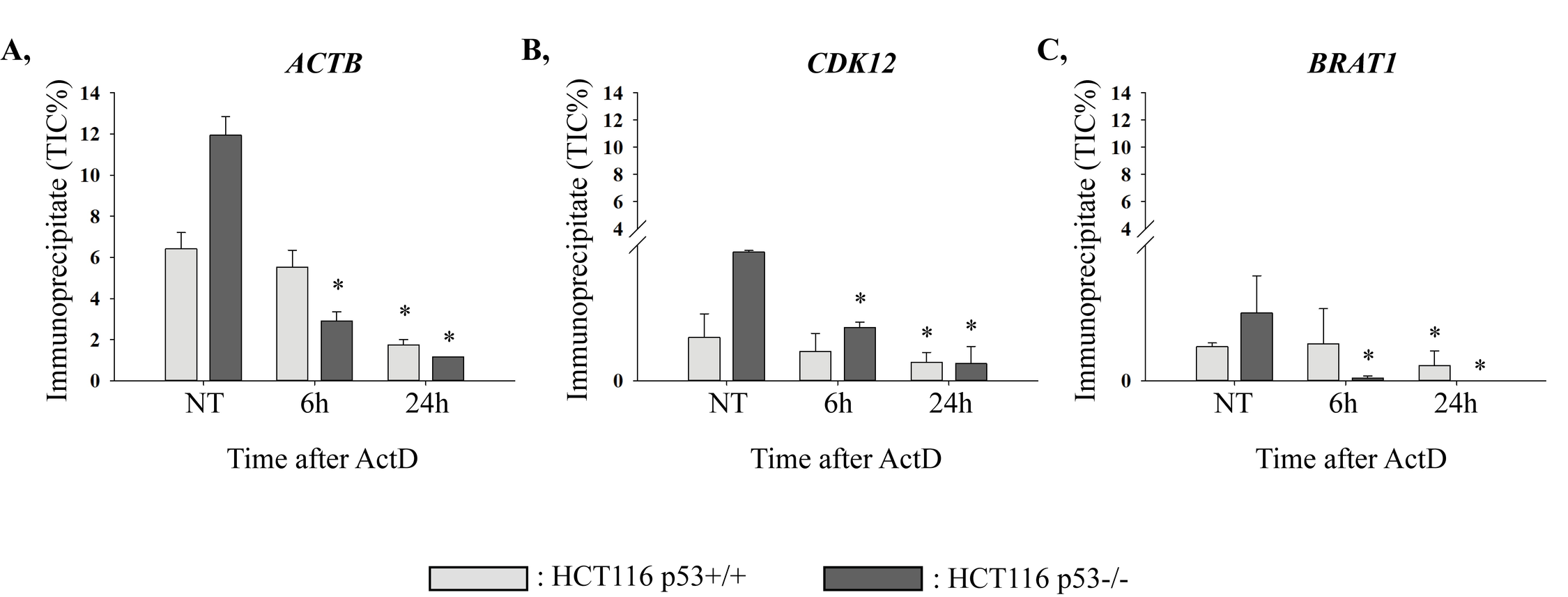

Supplement: S1 Fig — (TIF) [file pone.0267615.s003.tif]

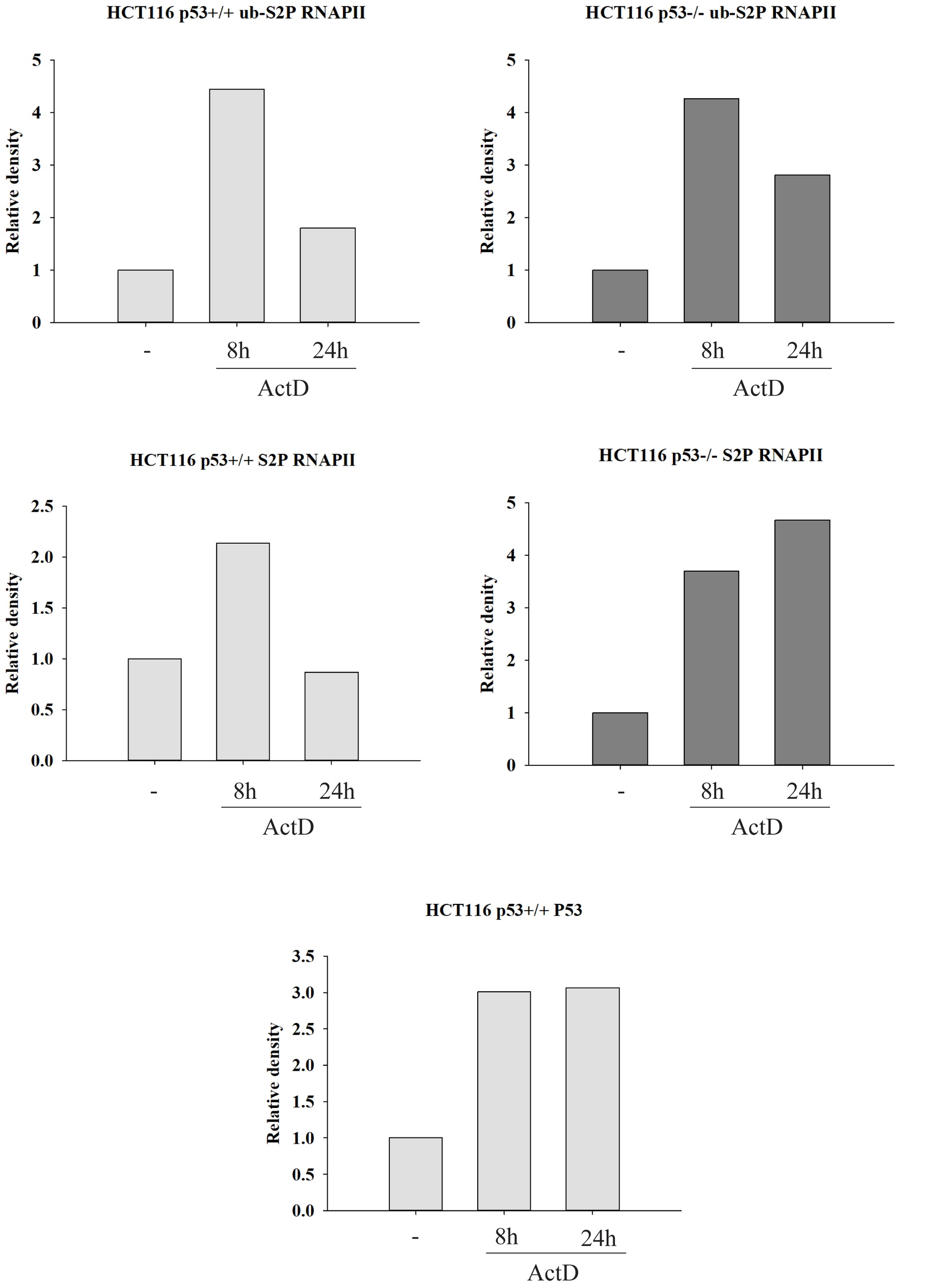

Supplement: S2 Fig — (TIF) [file pone.0267615.s004.tif]

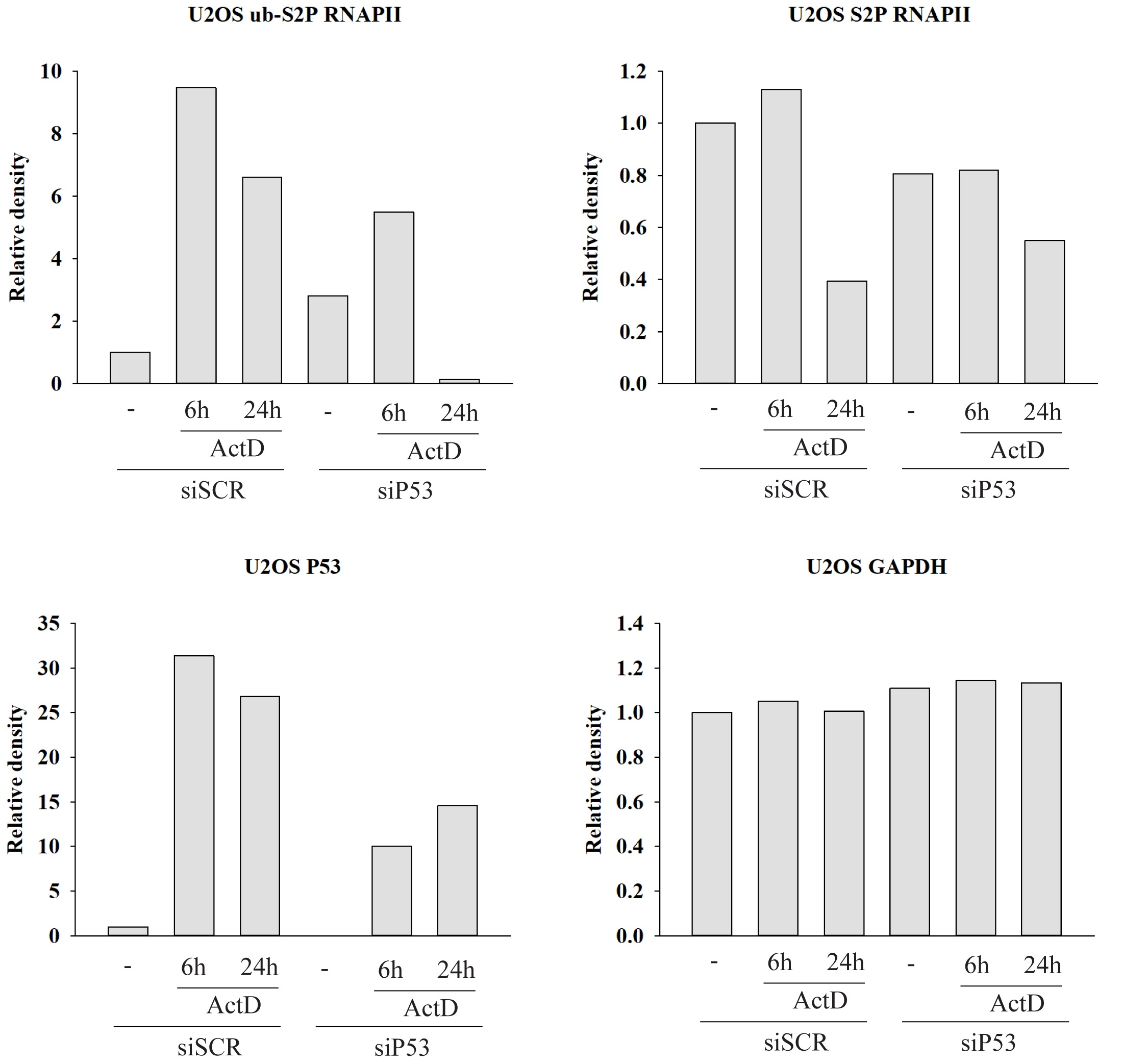

Supplement: S3 Fig — (TIF) [file pone.0267615.s005.tif]

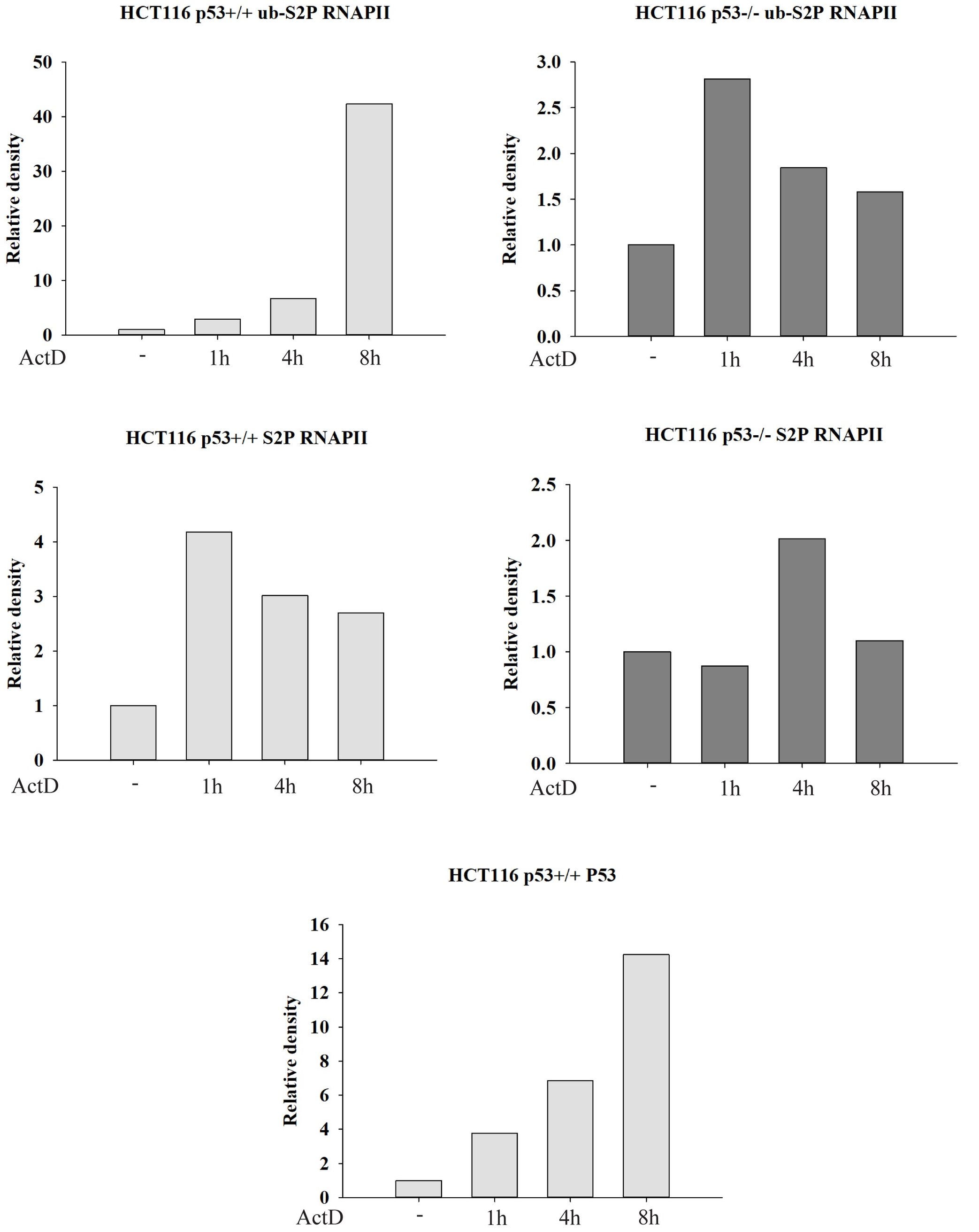

Supplement: S4 Fig — (TIF) [file pone.0267615.s006.tif]
