# Supplementary material for: The role of p53 in the DNA damage-related ubiquitylation of S2P RNAPII
Source: PLoS One. 2022 May 5;17(5):e0267615. doi: 10.1371/journal.pone.0267615 (PMC9070946; doi:10.1371/journal.pone.0267615)

Figure 2

Ub-S2P RNAPII

Figure 2A left panel HCT116 p53+/+

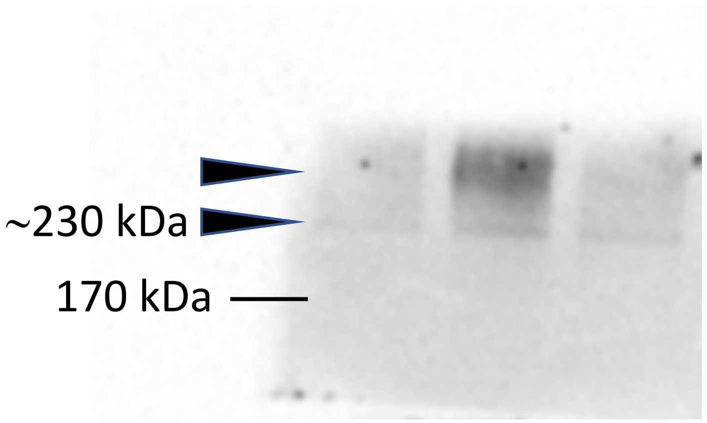

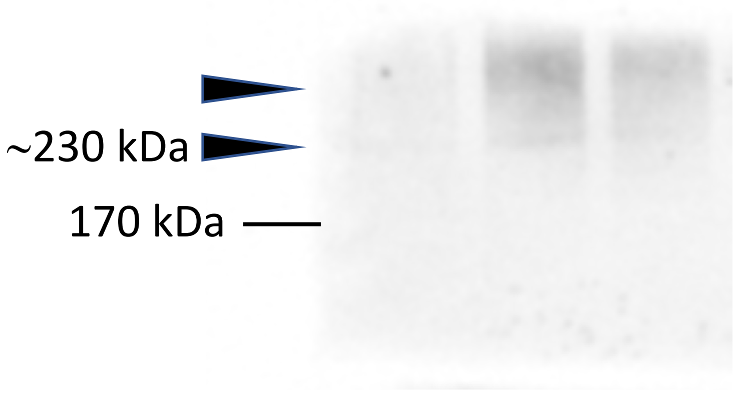

Figure 2A right panel HCT116 p53-/-

S2P RNAPII

Figure 2B left panel HCT116 p53+/+, first row

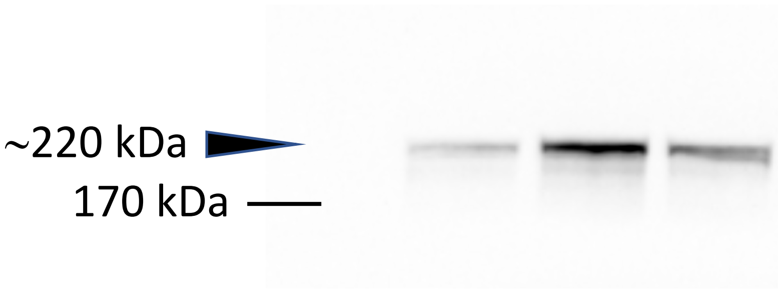

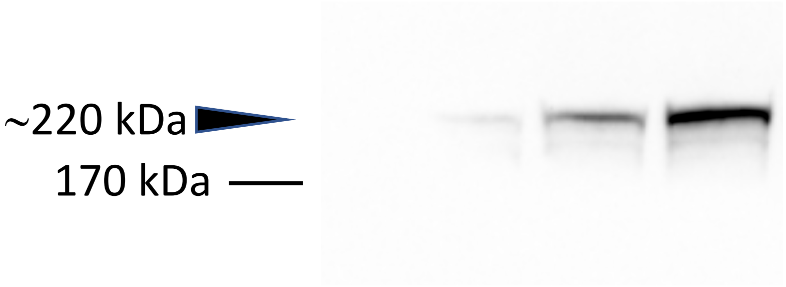

Figure 2B right panel HCT116 p53-/-, first row

P53

Figure 2B left panel HCT116 p53+/+, second row

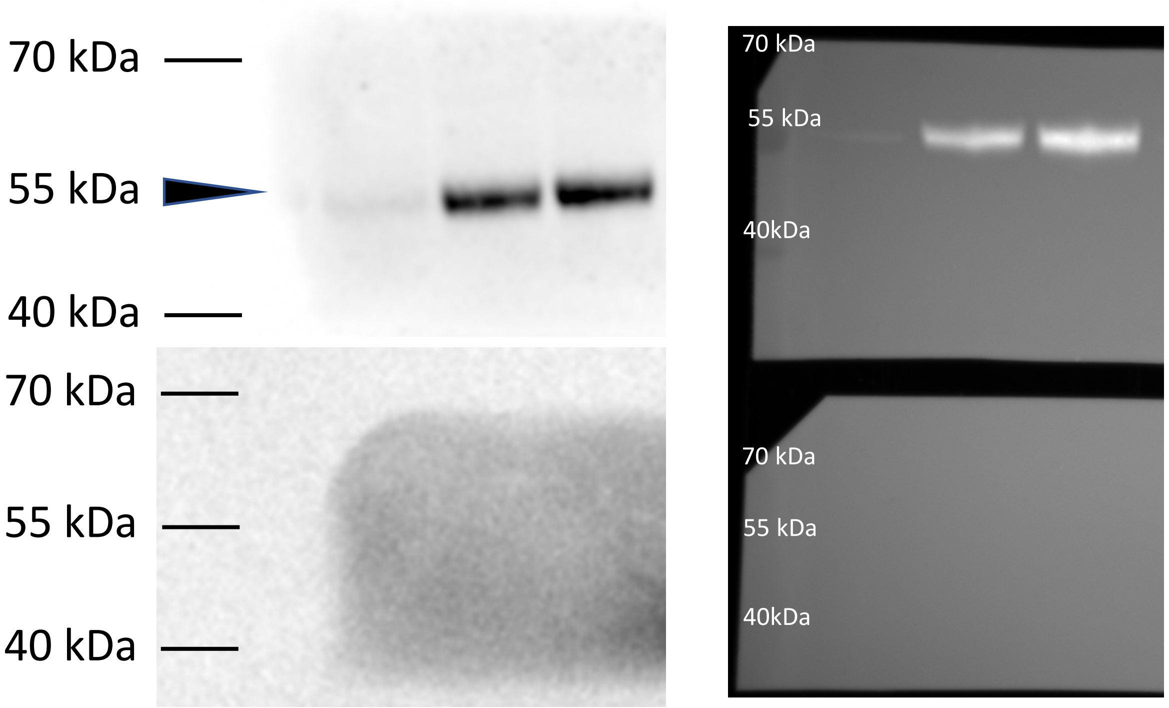

Figure 2B right panel HCT116 p53-/-, second row

Figure 2C, U2OS

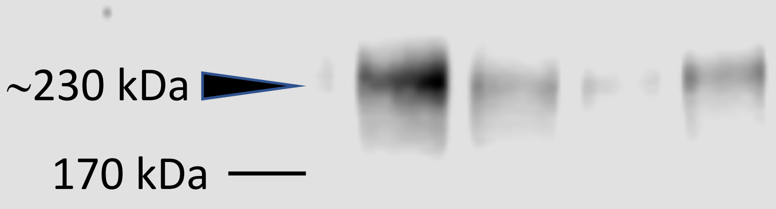

Figure 2D, first row, U2OS

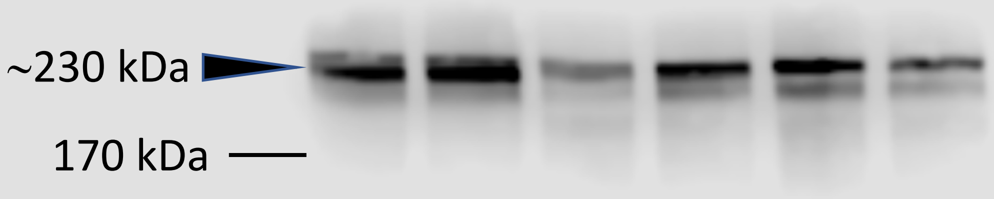

Figure 2D, second row, U2OS

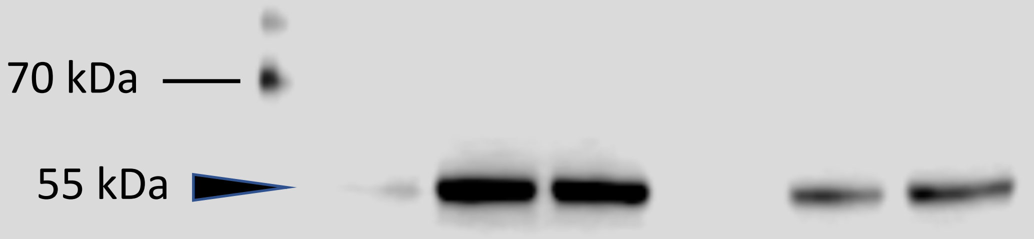

Figure 2D, third row, U2OS

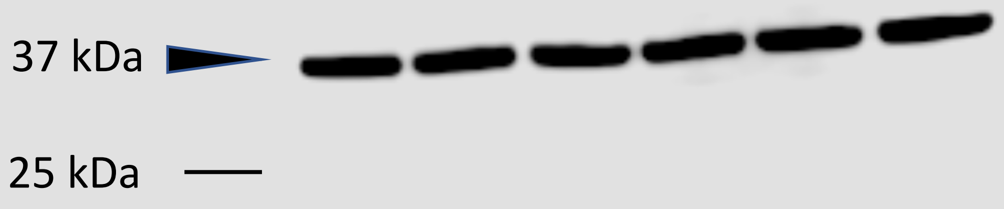

Figure 3

Ub-S2P RNAPII

Figure 3A

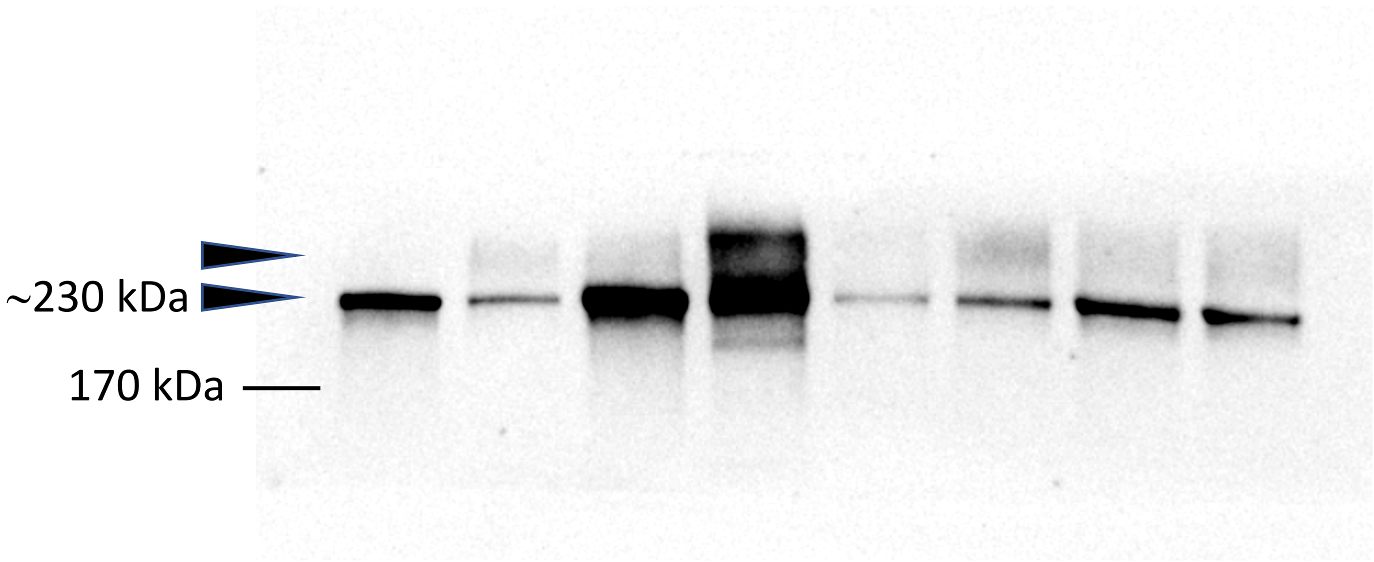

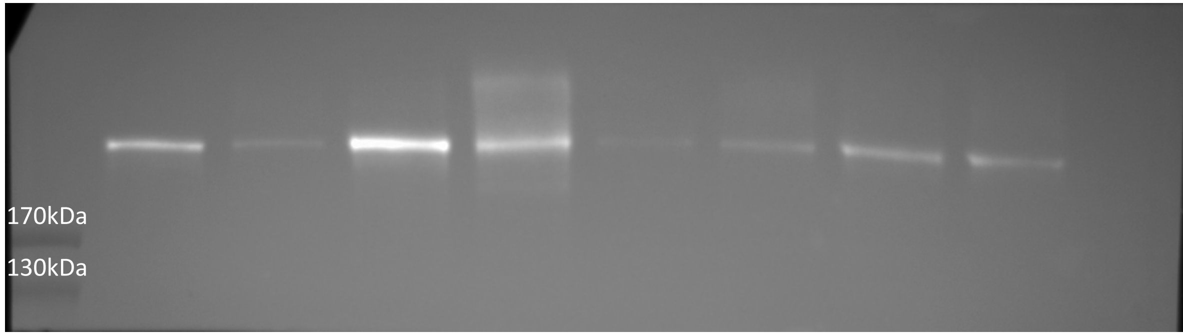

S2P RNAPII

Figure 3B, first row

~220 kDa

170 kDa

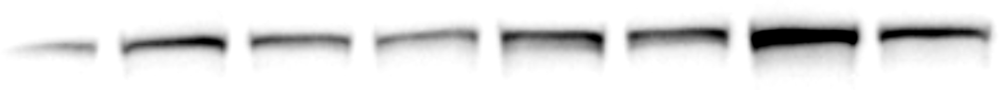

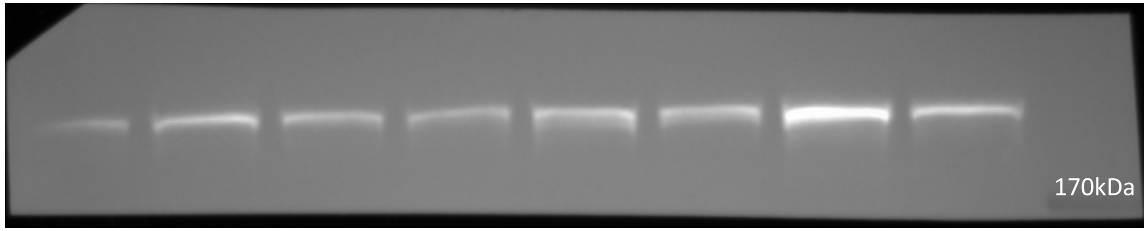

P53

Figure 3B, second row

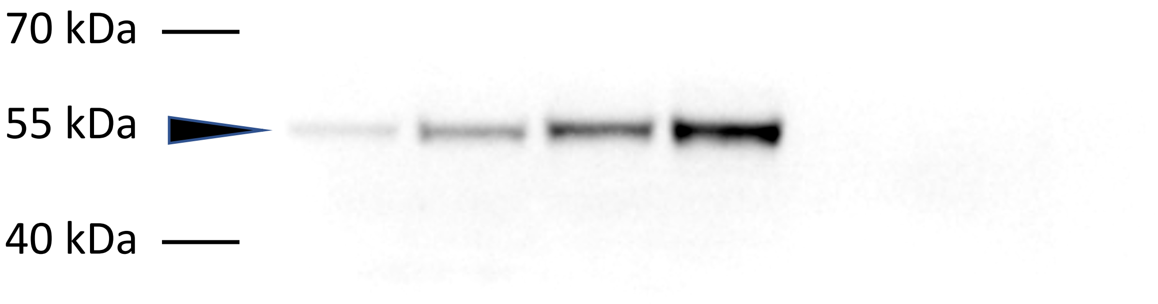

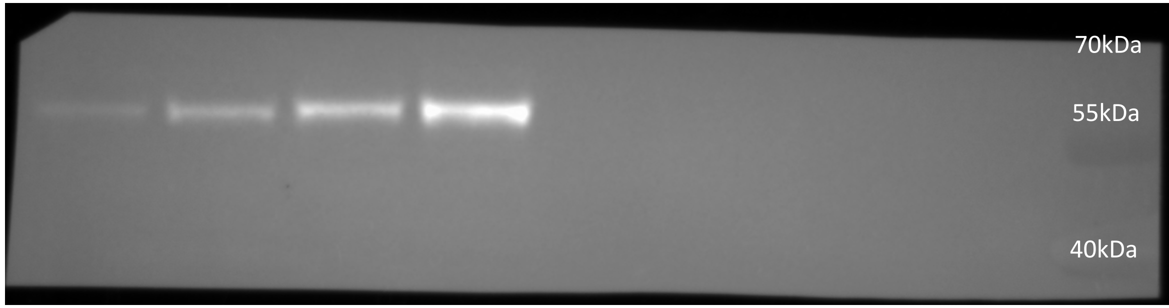

Supplement: S5 Fig — (PDF) [file pone.0267615.s007.pdf]
